# A phase I/II study of 10-min dosing of bendamustine hydrochloride (rapid infusion formulation) in patients with previously untreated indolent B-cell non-Hodgkin lymphoma, mantle cell lymphoma, or relapsed/refractory diffuse large B-cell lymphoma in Japan

**DOI:** 10.1007/s00280-022-04442-2

**Published:** 2022-07-07

**Authors:** Kenichi Ishizawa, Masahiro Yokoyama, Harumi Kato, Kazuhito Yamamoto, Masanori Makita, Kiyoshi Ando, Yasunori Ueda, Yoshimichi Tachikawa, Youko Suehiro, Mitsutoshi Kurosawa, Yoshihiro Kameoka, Hirokazu Nagai, Nobuhiko Uoshima, Takayuki Ishikawa, Michihiro Hidaka, Yoshikiyo Ito, Atae Utsunomiya, Koji Fukushima, Michinori Ogura

**Affiliations:** 1 Department of Hematology, Yamagata University Hospital, Yamagata, Japan; 2 Department of Hematology Oncology, The Cancer Institute Hospital of the Japanese Foundation for Cancer Research, Tokyo, Japan; 3 Department of Hematology and Cell Therapy, Aichi Cancer Center, Nagoya, Japan; 4 Department of Hematology, National Hospital Organization Okayama Medical Center, Okayama, Japan; 5 Department of Hematology and Oncology, Tokai University Hospital, Isehara, Japan; 6 Department of Hematology/Oncology, Ohara HealthCare Foundation Kurashiki Central Hospital, Kurashiki, Japan; 7 Department of Hematology, National Hospital Organization Kyushu Cancer Center, Fukuoka, Japan; 8 Department of Hematology, National Hospital Organization Hokkaido Cancer Center, Sapporo, Japan; 9 Department of Hematology, Akita University Hospital, Akita, Japan; 10 Department of Hematology, National Hospital Organization Nagoya Medical Center, Nagoya, Japan; 11 Department of Hematology, Japanese Red Cross Kyoto Daini Hospital, Kyoto, Japan; 12 Department of Hematology, Kobe City Medical Center General Hospital, Kobe, Japan; 13 Department of Hematology, National Hospital Organization Kumamoto Medical Center, Kumamoto, Japan; 14 Department of Hematology, Imamura General Hospital, Kagoshima, Japan; 15 SymBio Pharmaceuticals Limited, Tokyo, Japan; 16 Department of Hematology and Oncology, Kasugai Municipal Hospital, Kasugai, Japan

**Keywords:** Bendamustine, Rapid infusion, Non-Hodgkin lymphoma, Phase I/II, Safety, Tolerability

## Abstract

**Purpose:**

This phase I/II clinical study was conducted to examine the safety, tolerability, pharmacokinetics, and efficacy of 10-min dosing of bendamustine in patients with previously untreated indolent B-cell non-Hodgkin lymphoma (iNHL) or mantle cell lymphoma (MCL) (Group 1) and patients with relapsed/refractory diffuse large B-cell lymphoma (rrDLBCL) (Group 2).

**Methods:**

Rituximab 375 mg/m^2^ was administered intravenously every 28 days to Group 1 patients on day 1 and every 21 days to Group 2 patients on day 1. Bendamustine 90 mg/m^2^/day was administered to the former on days 1 and 2; bendamustine 120 mg/m^2^/day was administered to the latter on days 2 and 3. Each regimen was delivered up to six cycles for both groups. The primary endpoints were safety and tolerability in Groups 1 and 2, respectively.

**Results:**

Among 37 enrolled patients, safety was assessed in 36. In Group 1 (*n* = 30), 27 patients (90%) had follicular lymphoma. Adverse events (AEs) were observed in all 30 patients in Group 1. Dose-limiting toxicities were observed in two of six patients in Group 2. Common AEs included lymphocyte count decreased (86.7%, 100%). In Group 1, overall response and complete response rates were 93.1% (95% confidence interval [CI] 77.2–99.2%) and 75.9% (95% CI 56.5–89.7%), respectively. The *C*_max_ and AUC of bendamustine tended to be higher in Group 2 than in Group 1.

**Conclusions:**

This study showed that bendamustine is safe, well-tolerated and effective for patients with previously untreated iNHL, MCL or rrDLBCL. Pharmacokinetic data were equivalent to those obtained outside of Japan.

**Registration numbers:**

Registration NCT03900377; registered April 3, 2019.

**Supplementary Information:**

The online version contains supplementary material available at 10.1007/s00280-022-04442-2.

## Introduction

Bendamustine hydrochloride (BDM), synthesized in Germany in the 1960s, is an anticancer drug with alkylating and antimetabolite properties [1–4]. BDM has shown efficacy for hematologic malignancies and solid tumors [5–9].

The original formulation of BDM (original BDM) marketed in the United States was a product for 60-min infusion that was supplied as a lyophilized powder requiring reconstitution with sterile water to a 5 mg/mL solution before further dilution into a 500-mL infusion bag of either 0.9% sodium chloride injection (normal saline) or 2.5% dextrose/0.45% sodium chloride injection [10]. The maximum plasma concentration of bendamustine was achieved at the end of intravenous infusion (~ 1 h), followed by rapid elimination in a triphasic manner [11] and with an intermediate elimination half-life (*t*_1/2_) of ~ 40 min as the effective *t*_1/2_ [12].

In a phase I, open-label, randomized, crossover study [10], Cheung et al. compared original BDM with a new 10-min rapid infusion formulation (rapid BDM) supplied as a ready-to-dilute solution of 25 mg/mL. Consequently, the authors demonstrated the bioequivalence and comparable safety of original BDM and rapid BDM, and the Food and Drug Administration approved rapid BDM for the treatment of patients with chronic lymphocytic leukemia or indolent B-cell non-Hodgkin lymphoma (iNHL) that has progressed during or within 6 months of treatment with rituximab or a rituximab-containing regimen [13].

SymBio Pharmaceuticals Limited licensed rapid BDM from Eagle Pharmaceuticals, Inc. (Woodcliff Lake, NJ, USA) and gained regulatory approval for a 60-min infusion formulation not requiring reconstitution in September 2020. However, clinical data for Japanese patients were required to obtain regulatory approval to modify the dosage and administration to a 10-min infusion.

Based on the above, we conducted the present clinical phase I/II trial in Japanese B-cell lymphoma patients to examine the safety, efficacy, and tolerability of 10-min dosing of rapid BDM and the pharmacokinetics (PK) of bendamustine.

## Patients and methods

### Study design, endpoints, and procedures

The present clinical trial was a multicenter, open-label, phase I/II clinical study and consisted of two study groups. Group 1 comprised patients with previously untreated iNHL or mantle cell lymphoma (MCL) and Group 2 consisted of patients with relapsed/refractory diffuse large B-cell lymphoma (rrDLBCL). 375 mg/m^2^ of rituximab was administered intravenously on day 1 and every 28 days to Group 1 patients and every 21 days to Group 2 patients (on the day before day 1 of the first cycle for both groups), followed by bendamustine 90 mg/m^2^/day administered intravenously on days 1 and 2 for Group 1 and bendamustine 120 mg/m^2^/day on days 2 and 3 for Group 2. The primary endpoint was safety in Group 1 and tolerability in Group 2. Secondary endpoints were pharmacokinetics (PK) in Groups 1 and 2 and efficacy in Group 1.

The study period was from the time of obtaining informed consent of the patient to completion of the administration period, with follow-up monitoring once every 3 months for patients who received at least 1 dose of bendamustine through the 28-day cycles (the 29-day cycle for the first cycle only) in Group 1 and through the 21-day cycles in Group 2. The treatment period was up to 6 cycles. After cycle 2, the dose of bendamustine was reduced or study treatment postponed or discontinued on an as-needed basis according to the next cycle initiation criteria (e.g., neutrophil count: ≥ 1000/mm^3^) and the bendamustine dose reduction criteria in the second or subsequent cycles (e.g., grade 4 neutrophil count decreased [< 500/mm^3^] lasting for 1 or more weeks) based on treatment-emergent adverse events (TEAEs) found in the previous cycle and during follow-up.

### Patient eligibility

Eligibility criteria were as follows: (1) 20–79 years old; (2) survival expectancy of at least 3 months; (3) Eastern Cooperative Cancer Oncology Group performance status score of 0–2 [14]; (4) major organs presenting well-conserved function; (5) patient written informed consent; and (6) A—histopathologically confirmed, CD20-positive, iNHL (small lymphocytic lymphoma, splenic marginal zone lymphoma, lymphoplasmacytic lymphoma, extranodal marginal zone lymphoma of mucosa-associated lymphoid tissue, nodal marginal zone lymphoma, and follicular lymphoma (FL, grades 1, 2, 3a) or MCL (excluding transformed lymphoma) [15]; B—a measurable lesion; C—absence of treatment history; and D—at least one of the criteria listed in GELF (excluding MCL patients) [16–18] in Group 1; and (7) A—CD20 positive, diffuse large B-cell lymphoma (excluding transformed lymphoma) [15] and B—rrDLBCL after R-CHOP (like) regimen as a first line treatment in Group 2.

The key exclusion criteria were as follows: (1) criteria common to Groups 1 and 2 were invasion into the central nervous system, patients with serious active infection requiring antibiotic, antifungal, or antiviral IV injection, and patients with serious complications, such as hepatic failure or renal failure; (2) the criterion specific to Group 1 was MCL patient ≤ 65 years of age; and (3) the criteria specific to Group 2 were a non-treatment period of < 3 weeks between the last day of previous treatment for DLBCL and enrollment and a history of allogeneic hematopoietic stem cell transplantation.

Between April 1, 2019, and September 9, 2020, the present study was conducted in Japan according to the provisions of the Declaration of Helsinki, Good Clinical Practice, and related regulations and protocols. All patients provided Institutional Review Board-approved written informed consent prior to the execution of any study-specific procedures or assessments. The present study was registered at ClinicalTrials.gov (NCT03900377).

### Safety and tolerability

Safety was assessed in all patients based on TEAEs (type, incidence, and severity) that were expressed according to Medical Dictionary for Regulatory Activities-Japanese (MedDRA-J) version 23.1, with grading defined according to the National Cancer Institute Common Terminology Criteria for Adverse Events version 4.0-Japan Clinical Oncology Group, as well as on time-course changes in laboratory values. The number of patients who developed dose-limiting toxicities (DLTs) was examined only in Group 2. DLTs were defined as follows: grade 4 neutrophil count decreased [< 500/mm^3^] lasting for 1 or more weeks with a fever of ≥ 38 °C; decreased platelet count [< 10,000/mm^3^] or a bleeding tendency requiring platelet transfusion; other grade 4 hematologic toxicities excluding lymphocyte count decreased and differential white blood counts (%); and other ≥ grade 3 nonhematologic toxicities.

### Blood sampling for pharmacokinetic studies

For six patients in Group 1 and all six patients in Group 2, blood sampling for PK of bendamustine was performed on day 1 of cycle 1 in Group 1 and day 2 of cycle 1 in Group 2 at the following ten timepoints: within 30 min before infusion initiation; at 5 and 10 min after infusion initiation; and at 5, 15, and 30 min and 1, 2, 4, and 6 h after infusion completion. Plasma concentrations of bendamustine were measured by high-performance liquid chromatograph–tandem mass spectrometer (LC–MS/MS) with using the analytical method validated by CMIC Pharma Science Co., Ltd. to calculate the following summary statistics according to noncompartmental model analysis using Phoenix WinNonlin version 6.4 (Certara, Princeton, NJ, USA): maximum concentration (*C*_max_), time of maximum observed concentration (*t*_max_), area under the concentration–time curve from the time of dosing to the time of the last measurable (positive) concentration (AUC_0-last_), area under the concentration–time curve from time of dosing extrapolated to infinity (AUC_0-inf_), and elimination half-life (*t*_1/2_).

### Efficacy

Efficacy was assessed in accordance with the following response criteria listed in the revised response criteria for malignant lymphoma (2007) [19]: complete response (CR) rate, overall response rate (ORR): CR + partial response (PR) rate, and progression-free survival (PFS).

### Statistical analyses

As our previous phase II study in 69 Japanese patients with previously untreated iNHL or MCL indicated grade 3–4 TEAEs mostly at incidence rates of 10–100% [20], the target number of patients in Group 1 was set to 30 as the sample size with ≥ 95% power to detect a TEAE (incidence: ≥ 10%). The target number of patients in Group 2 was set to 6 in reference to the previously amended Japanese version of the guidelines for clinical evaluation of anticancer drugs [21]. For analysis of efficacy, the best responses were evaluated for the CR rate and the ORR with binomial probability-based 95% confidence intervals [CIs]. Kaplan–Meier estimates were obtained to analyze PFS, with 50% points and 95% CIs according to the Greenwoods formula. Safety was analyzed in the safety population comprising enrolled patients who received at least one dose of bendamustine. The data sets generated during and/or analysed during the current study are not publicly available for confidentiality reasons but certain information may be available from the corresponding author upon request.

## Results

### Patient characteristics

**Figure 1:**
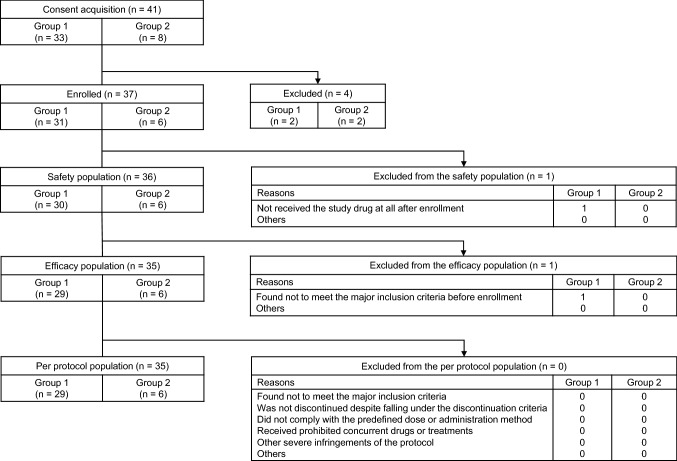
**Fig. 1 **Patient disposition

A total of 37 patients were enrolled, 36 and 35 of whom were assessed for safety and efficacy, respectively (Fig. 1). Thirty patients in Group 1 had histopathologically confirmed, CD20-positive, iNHL, and all six patients in Group 2 had rrDLBCL; the patients in this safety population had median ages of 67 years (range 43–76 years) and 73 years (range 69–78 years), respectively (Table 1). In Group 1, 27 patients (90%) had FL, 16 (53%) presented clinical stage IV, and 13 (48%) were categorized as being in the “high risk” category in the Follicular Lymphoma International Prognostic Index (FLIPI); 6 patients were assessed for pharmacokinetics as well. Four (67%) of 6 patients in Group 2 presented with clinical stage III, all 6 (100%) were responders to prior treatments, and 4 (67%) were categorized as being in the “low-intermediate risk” category in the International Prognostic Index (IPI).

**Table 1:** Demographic and patient characteristics in the safety population

Characteristics	Group 1 (*N* = 30) *n* (%)	Group 2 (*N* = 6) *n* (%)
*Sex*		
Male	12 (40)	3 (50)
Female	18 (60)	3 (50)
*Age—median years (range)*	67 (43–76)	73 (69–78)
<65	10 (33)	0 (0)
≥65	20 (67)	6(100)
*Diagnosis (WHO classification)*		
Extranodal marginal zone lymphoma of mucosa-associated lymphoid tissue	1 (3)	–
Nodal marginal zone B-cell lymphoma	1(3)	–
Follicular lymphoma	27 (90)	–
Mantle cell lymphoma	1(3)	–
Diffuse large B-cell lymphoma	–	6(100)
*Cell-of-origin*		
Germinal center B-cell-like (GCB)	–	2 (33)
Non-GCB	–	4 (67)
*Clinical stage (Ann Arbor classification)*		
I	2(7)	0(0)
II	6(20)	1(17)
III	6(20)	4(67)
IV	16(53)	1(17)
Unknown	0(0)	0(0)
*History of primer treatments*		
Absent	30(100)	0(0)
Present	0(0)	6(100)
*Lines of prior treatments median (range)*		2(1–6)
1 regimen	–	1(17)
2 regimens	–	4(67)
≥3 regimens	–	1(17)
*Response to prior treatments*^a^		
Responder	–	6(100)
Nonresponder	–	0(0)
Unknown	–	0(0)
*Autologous hematopoietic stem cell transplantation*		
Absent	–	6(100)
Present	–	0(0)
Radiotherapy		
Absent	–	5 (83)
Present	–	1(17)
*Performance status (ECOG criteria)*		
0	25(83)	4(67)
1	5(17)	2(33)
2	0(0)	0(0)
3	0(0)	0(0)
4	0(0)	0(0)
*Systemic symptoms (B symptoms) (Ann Arbor classification)*^b^		
Absent	17(57)	4(67)
Present	13(43)	2(33)
Unknown	0(0)	0(0)
Tumor diameter		
<5 cm	10(33)	–
≥5 cm	20(67)	–
LDH		
≤ Upper limit of normal	22(73)	5(83)
> Upper limit of normal	8(27)	1(17)
*Number of nodal lesions*^c^		
<5	19(63)	6(100)
≥5	11(37)	0(0)
*Number of extranodal lesions*^d^		
<2	24(80)	6(100)
≥2	6(20)	0(0)
Bone marrow infiltration		
Present	11(37)	0(0)
Absent	19(64)	6(100)
Undetermined	0(0)	0(0)
Unknown	0(0)	0(0)
*FLIPI risk category for FL*^e^	27(100)	–
Low (score: 0–1)	7(26)	–
Intermediate (score: 2)	7(26)	–
High (poor) (score: 3–5)	13(48)	–
Unknown	0(0)	–
*IPI risk category*^f^		
Low (score: 0–1)	–	1(17)
Low–intermediate (score: 2)	–	4(67)
High–intermediate (score: 3)	–	1(17)
High (score: 4–5)	–	0(0)
Unknown	–	0(0)

–: not applicable

*N* number of patients, *ECOG* Eastern Clinical Oncology Group, *LDH* lactate dehydrogenase, *FLIPI* follicular lymphoma international prognostic index, *FL* follicular lymphoma, *IPI* international prognostic index

^a^The patient, whose best response to 1 or more prior treatments was categorized as “CR or PR”, was categorized as “responder”

^b^Systemic symptoms (B symptoms): 1 or more tumor-related symptoms were found prior to the initiation of administration

^c^Number of nodal lesions: the sum of the number of nodal target lesions and nodal non-target lesions

^d^Number of extranodal lesions: the sum of the number of extranodal non-target lesions, as well as of the cases of hepatomegaly, renal enlargement, and bone marrow infiltration

^e^Categorized based on the number of corresponding poor prognostic factors: age, ≥ 61 years; LDH, > upper limit of normal; hemoglobin, < 12 g/dL; number of nodal lesions, ≥ 5; and clinical stage, III or IV

^f^Categorized based on the number of corresponding poor prognostic factors: age, ≥ 61 years; LDH, > upper limit of normal; performance status, 2–4; clinical stage, III or IV; and the number of extranodal lesions, ≥ 2

### Exposure

The median number of delivered cycles in Groups 1 and 2 was 6 [range 1–6] and 2.5 [range 1–5], respectively. In Group 1, 18 patients received a maximum of 6 cycles. In Group 2, 1 patient received a maximum of 5 cycles, but none of the 6 patients completed 6 cycles. Major causes of treatment discontinuation in Groups 1 and 2 were failure to meet the next cycle initiation criteria (16.7% and 50.0%, respectively), TEAEs (6.7% and 16.7%, respectively), and other causes, including disease progression (13.3% and 33.3%, respectively).

### Safety

TEAEs (incidence: ≥ 10%) in the safety population are summarized in Table 2. In Group 1, the most common hematologic TEAEs were decreased lymphocyte (87%), neutrophil and leukocyte (83% each) counts as well as decreased CD4 lymphocyte counts (77%). Grade 3/4 lymphocyte count decreased occurred in 87% of patients, while grade 3/4 neutrophil count decreased, grade 3/4 white blood cell count decreased, and grade 3/4 CD4 lymphocytes decreased occurred in 77% each of patients. Among nonhematologic TEAEs, nausea (73%), infusion-related reaction (63%), and constipation (50%) were most common. Serious TEAEs occurred in 13% of patients: 1 case each of febrile neutropenia, infection, cytomegalovirus enterocolitis, and infusion-related reaction. All these serious TEAEs were resolved or alleviated. TEAEs leading to treatment discontinuation occurred in 20% of patients, and TEAEs leading to dose reduction occurred in 13.3%. Grade 3 or greater laboratory value abnormalities occurred in 90% of patients.

**Table 2:** Summary of TEAEs (incidence: ≥ 10%) in the safety population

	Patients in group 1 (*n* = 30), group 2 (*n* = 6)						
	All Grades, *n* (%)	Grade, *n* (%)					Grades 3–5 *n* (%)
		1	2	3	4	5	
*Group 1 Hematologic*							
Lymphocyte count decreased	26 (87)	0 (0)	0 (0)	2 (7)	24 (80)	0 (0)	26 (87)
Neutrophil count decreased	25 (83)	0 (0)	2 (7)	12 (40)	11 (37)	0 (0)	23 (77)
White blood cell count decreased	25 (83)	0 (0)	2 (7)	18 (60)	5 (17)	0 (0)	23 (77)
CD4 lymphocytes decreased	23 (77)	0 (0)	0 (0)	9 (30)	14 (47)	0 (0)	23 (77)
Platelet count decreased	14 (47)	12 (40)	2 (7)	0 (0)	0 (0)	0 (0)	0 (0)
Anaemia	10 (33)	4 (13)	5 (17)	1 (3)	0 (0)	0 (0)	1 (3)
Neutropenia	3 (10)	0 (0)	0 (0)	1 (3)	2 (7)	0 (0)	3 (10)
*Group 1 Nonhematologic*							
Nausea	22 (73)	14 (47)	7 (23)	1 (3)	0 (0)	0 (0)	1 (3)
Infusion related reaction	19 (63)	4 (13)	14 (47)	1 (3)	0 (0)	0 (0)	1 (3)
Constipation	15 (50)	9 (30)	6 (20)	0 (0)	0 (0)	0 (0)	0 (0)
Malaise	14 (47)	13 (43)	1 (3)	0 (0)	0 (0)	0 (0)	0 (0)
Decreased appetite	11 (37)	9 (30)	1 (3)	1 (3)	0 (0)	0 (0)	1 (3)
ALT increased	9 (30)	4 (13)	2 (7)	3 (10)	0 (0)	0 (0)	3 (10)
Rash	9 (30)	6 (20)	3 (10)	0 (0)	0 (0)	0 (0)	0 (0)
AST increased	8 (27)	5 (17)	2 (7)	1 (3)	0 (0)	0 (0)	1 (3)
Gamma-glutamyltransferase increased	8 (27)	1 (3)	2 (7)	5 (17)	0 (0)	0 (0)	5 (17)
Diarrhoea	8 (27)	5 (17)	3 (10)	0 (0)	0 (0)	0 (0)	0 (0)
Blood immunoglobulin M decreased	7 (23)	7 (23)	0 (0)	0 (0)	0 (0)	0 (0)	0 (0)
C-reactive protein increased	7 (23)	7 (23)	0 (0)	0 (0)	0 (0)	0 (0)	0 (0)
Hepatic function abnormal	6 (20)	3 (10)	3 (10)	0 (0)	0 (0)	0 (0)	0 (0)
Vomiting	6 (20)	5 (17)	1 (3)	0 (0)	0 (0)	0 (0)	0 (0)
Blood lactate dehydrogenase increased	6 (20)	6 (20)	0 (0)	0 (0)	0 (0)	0 (0)	0 (0)
Headache	6 (20)	4 (13)	2 (7)	0 (0)	0 (0)	0 (0)	0 (0)
Taste disorder	6 (20)	6 (20)	0 (0)	0 (0)	0 (0)	0 (0)	0 (0)
Phlebitis	6 (20)	0 (0)	6 (20)	0 (0)	0 (0)	0 (0)	0 (0)
Pyrexia	5 (17)	5 (17)	0 (0)	0 (0)	0 (0)	0 (0)	0 (0)
Blood immunoglobulin G decreased	5 (17)	4 (13)	1 (3)	0 (0)	0 (0)	0 (0)	0 (0)
Blood alkaline phosphatase increased	5 (17)	4 (13)	1 (3)	0 (0)	0 (0)	0 (0)	0 (0)
Insomnia	5 (17)	5 (17)	0 (0)	0 (0)	0 (0)	0 (0)	0 (0)
Blood albumin decreased	4 (13)	2 (7)	2 (7)	0 (0)	0 (0)	0 (0)	0 (0)
Pruritis	4 (13)	2 (7)	2 (7)	0 (0)	0 (0)	0 (0)	0 (0)
Vascular pain	4 (13)	4 (13)	0 (0)	0 (0)	0 (0)	0 (0)	0 (0)
Beta 2 microglobulin increased	3 (10)	3 (10)	0 (0)	0 (0)	0 (0)	0 (0)	0 (0)
Blood immunoglobulin A decreased	3 (10)	3 (10)	0 (0)	0 (0)	0 (0)	0 (0)	0 (0)
Electrocardiogram QT prolonged	3 (10)	2 (7)	1 (3)	0 (0)	0 (0)	0 (0)	0 (0)
Protein total decreased	3 (10)	3 (10)	0 (0)	0 (0)	0 (0)	0 (0)	0 (0)
Hypoalbuminaemia	3 (10)	3 (10)	0 (0)	0 (0)	0 (0)	0 (0)	0 (0)
Back pain	3 (10)	2 (7)	1 (3)	0 (0)	0 (0)	0 (0)	0 (0)
Dry skin	3 (10)	2 (7)	1 (3)	0 (0)	0 (0)	0 (0)	0 (0)
Erythema	3 (10)	2 (7)	1 (3)	0 (0)	0 (0)	0 (0)	0 (0)
*Group 2 Hematologic*							
Lymphocyte count decreased	6 (100)	0 (0)	0 (0)	0 (0)	6 (100)	0 (0)	6 (100)
White blood cell decreased	6 (100)	0 (0)	3 (50)	2 (33)	1 (17)	0 (0)	3 (50)
Neutrophil count decreased	5 (83)	1 (17)	2 (33)	1 (17)	1 (17)	0 (0)	2 (33)
Platelet count decreased	5 (83)	2 (33)	0 (0)	3 (50)	0 (0)	0 (0)	3 (50)
Anaemia	3 (50)	0 (0)	2 (33)	1 (17)	0 (0)	0 (0)	1 (17)
CD4 lymphocytes decreased	3 (50)	0 (0)	0 (0)	2 (33)	1 (17)	0 (0)	3 (50)
White blood cell count increased	1 (17)	1 (17)	0 (0)	0 (0)	0 (0)	0 (0)	0 (0)
Neutrophil count increased	1 (17)	1 (17)	0 (0)	0 (0)	0 (0)	0 (0)	0 (0)
Red blood cell count decreased	1 (17)	1 (17)	0 (0)	0 (0)	0 (0)	0 (0)	0 (0)
*Group 2 Nonhematologic*							
Nausea	5 (83)	4 (67)	0 (0)	1 (17)	0 (0)	0 (0)	1 (17)
Malaise	4 (67)	4 (67)	0 (0)	0 (0)	0 (0)	0 (0)	0 (0)
Decreased appetite	4 (67)	3 (50)	0 (0)	1 (17)	0 (0)	0 (0)	1 (17)
Stomatitis	2 (33)	1 (17)	1 (17)	0 (0)	0 (0)	0 (0)	0 (0)
Vomiting	2 (33)	1 (17)	0 (0)	1 (17)	0 (0)	0 (0)	1 (17)
Pyrexia	2 (33)	1 (17)	1 (17)	0 (0)	0 (0)	0 (0)	0 (0)
Fall	2 (33)	2 (33)	0 (0)	0 (0)	0 (0)	0 (0)	0 (0)
Infusion related reaction	2 (33)	1 (17)	1 (17)	0 (0)	0 (0)	0 (0)	0 (0)
AST increased	2 (33)	1 (17)	1 (17)	0 (0)	0 (0)	0 (0)	0 (0)
Insomnia	2 (33)	1 (17)	1 (17)	0 (0)	0 (0)	0 (0)	0 (0)
Rash	2 (33)	1 (17)	1 (17)	0 (0)	0 (0)	0 (0)	0 (0)
Abdominal pain	1 (17)	0 (0)	1 (17)	0 (0)	0 (0)	0 (0)	0 (0)
Constipation	1 (17)	1 (17)	0 (0)	0 (0)	0 (0)	0 (0)	0 (0)
Ileus	1 (17)	0 (0)	0 (0)	1 (17)	0 (0)	0 (0)	1 (17)
Subileus	1 (17)	0 (0)	0 (0)	1 (17)	0 (0)	0 (0)	1 (17)
Folliculitis	1 (17)	0 (0)	1 (17)	0 (0)	0 (0)	0 (0)	0 (0)
Oral candidiasis	1 (17)	0 (0)	1 (17)	0 (0)	0 (0)	0 (0)	0 (0)
Pharyngitis	1 (17)	0 (0)	1 (17)	0 (0)	0 (0)	0 (0)	0 (0)
Rib fracture	1 (17)	0 (0)	1 (17)	0 (0)	0 (0)	0 (0)	0 (0)
ALT increased	1 (17)	1 (17)	0 (0)	0 (0)	0 (0)	0 (0))	0 (0)
Blood creatinine increased	1 (17)	0 (0)	1 (17)	0 (0)	0 (0)	0 (0)	0 (0)
Blood immunoglobulin G decreased	1 (17)	0 (0)	1 (17)	0 (0)	0 (0)	0 (0)	0 (0)
Blood immunoglobulin M decreased	1 (17)	0 (0)	1 (17)	0 (0)	0 (0)	0 (0)	0 (0)
Gamma-glutamyltransferase increased	1 (17)	1 (17)	0 (0)	0 (0)	0 (0)	0 (0)	0 (0)
Weight decreased	1 (17)	0 (0)	0 (0)	1 (17)	0 (0)	0 (0)	1 (17)
Hepatitis B DNA assay positive	1 (17)	1 (17)	0 (0)	0 (0)	0 (0)	0 (0)	0 (0)
Diabetes mellitus	1 (17)	0 (0)	1 (17)	0 (0)	0 (0)	0 (0)	0 (0)
Disseminated intravascular coagulation	1 (17)	0 (0)	0 (0)	1 (17)	0 (0)	0 (0)	1 (17)
Folate deficiency	1 (17)	0 (0)	1 (17)	0 (0)	0 (0)	0 (0)	0 (0)
Hypoalbuminaemia	1 (17)	1 (17)	0 (0)	0 (0)	0 (0)	0 (0)	0 (0)
Hyponatraemia	1 (17)	0 (0)	0 (0)	1 (17)	0 (0)	0 (0)	1 (17)
Dizziness	1 (17)	1 (17)	0 (0)	0 (0)	0 (0)	0 (0)	0 (0)
Headache	1 (17)	0 (0)	1 (17)	0 (0)	0 (0)	0 (0)	0 (0)
Proteinuria	1 (17)	0 (0)	1 (17)	0 (0)	0 (0)	0 (0)	0 (0)
Acute respiratory failure	1 (17)	0 (0)	0 (0)	0 (0)	0 (0)	1 (17)	1 (17)
Cough	1 (17)	0 (0)	1 (17)	0 (0)	0 (0)	0 (0)	0 (0)
Epistaxis	1 (17)	1 (17)	0 (0)	0 (0)	0 (0)	0 (0)	0 (0)
Hiccups	1 (17)	1 (17)	0 (0)	0 (0)	0 (0)	0 (0)	0 (0)
Dermatitis acneiform	1 (17)	0 (0)	1 (17)	0 (0)	0 (0)	0 (0)	0 (0)
Erythema	1 (17)	1 (17)	0 (0)	0 (0)	0 (0)	0 (0)	0 (0)
Vascular pain	1 (17)	1 (17)	0 (0)	0 (0)	0 (0)	0 (0)	0 (0)
Vasculitis	1 (17)	0 (0)	1 (17)	0 (0)	0 (0)	0 (0)	0 (0)

*n* number of patients, *TEAEs* treatment-emergent adverse events, *ALT* alanine aminotransferase, *AST* aspartate aminotransferase

In Group 2, the most common hematologic TEAEs were decreased lymphocyte count and decreased white blood cell count (100% each) as well as decreased neutrophil count and decreased platelet count (83% each). Grade 3/4 lymphocyte count decreased occurred in 100% of patients. In contrast, grade 3/4 white blood cell count decreased and grade 3/4 platelet count decreased occurred in 50% each of patients, and grade 3/4 neutrophil count decreased occurred in 33% of patients. Among nonhematologic TEAEs, nausea (83%) and malaise and decreased appetite (67%) were most common. DLTs occurred in two patients: 1 case each of grade 3 vomiting and nausea. These DLTs quickly reversed, and no concerns about the tolerability of bendamustine arose, because those TEAEs were considered manageable with antiemetic prophylaxis or treatment. Seven cases of serious TEAEs occurred in 50% of patients. With the exception of decreased appetite and acute respiratory failure in one patient who died, all these events reversed.

No deaths occurred before completion of the bendamustine dosing period. Two patients with FL in Group 1 died after completion of the study: one due to pneumocystis pneumonia and the other due to primary disease progression. In addition, three patients with DLBCL in Group 2 died after study completion: 1 patient due to acute respiratory failure and two patients due to primary disease progression.

### Efficacy

The best overall responses in the patients analyzed for efficacy are summarized in Table 3. In Group 1, the CR rate [19] was 75.9% (95% CI 56.5–89.7%), and the ORR was 93.1% (95% CI 77.2–99.2%). The median PFS was not reached within the median follow-up period of 275.0 days (52–481 days). ORRs were not greatly influenced by the number of delivered cycles. In Group 2, CR and OR rates were 50.0% (95% CI 11.8–88.2%) and 66.7% (95% CI 22.3–95.7%), respectively.

**Table 3:** Best overall responses in patients analyzed for efficacy

	Group 1 (*N* = 29) *n* (%)	Group 2 (*N* = 6) *n* (%)
*Best overall response*^a^		
CR	22 (75.9)	3 (50.0)
PR	5 (17.2)	1 (16.7)
SD	1 (3.4)	0 (0.0)
PD	1 (3.4)	2 (33.3)
NE	0 (0.0)	0 (0.0)
ORR^b^	27 (93.1)	4 (66.7)
95% CI, %^c^	77.2–99.2	22.3–95.7
CR rate	22 (75.9)	3 (50.0)
95% CI, %^c^	56.5–89.7	11.8–88.2

*N* number of patients, *CR* complete response, *PR* partial response, *SD* stable disease, *PD* progressive disease, *NE* not evaluable, *ORR* overall response rate, *CI* confidence interval

^a^Assessed in accordance with the revised response criteria for malignant lymphoma (Cheson et al. J Clin Oncol. 2007;25(5):579–86)

^b^The number and rate of patients who were categorized as CR or PR

^c^The precise 95% confidence interval based on binominal probability

### Pharmacokinetic analyses in Groups 1 and 2

**Figure 2:**
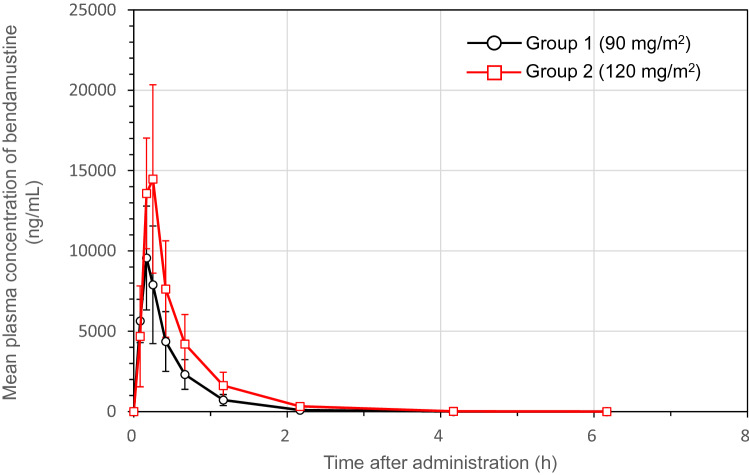
**Fig. 2 **Time-course changes in plasma bendamustine concentration. In both Group 1 (open circles) and Group 2 (open squares), maximum plasma concentrations of bendamustine were achieved at approximately 10 min after administration, coinciding with the completion of administration. A rapid triphasic decline occurred thereafter

The *C*_max_, AUC_0-last_, and AUC_0-inf_ of bendamustine tended to be higher in Group 2 than in Group 1: *C*_max_ (Group1 Mean; 9809 ng/mL, Group 2 Mean; 16256 ng/mL), AUC_0-last_ (4707 ng·h/mL, 8242 ng·h/mL, respectively) and AUC_0-inf_ (4708 ng·h/mL, 8244 ng·h/mL, respectively), though its *t*_max_ and *t*_1/2_ were similar in both groups (Table 4). Mean plasma concentrations of bendamustine in Groups 1 and 2 peaked at the end of the 10-min infusion (Fig. 2), followed by a rapid triphasic decline, as observed in the phase I clinical trial [3]. The pharmacokinetic parameters (*C*_max_, *t*_max_, AUC_0-last_, AUC_0-inf_, and *t*_1/2_) of bendamustine 120 mg/m^2^ were comparable between the present study and the study conducted by Cheung et al. [10] (Supplementary Table 1).

**Table 4:** Summary statistics of the major pharmacokinetic parameters of bendamustine in patients analyzed for pharmacokinetics

Group (dose)	*C*_max_ (ng/mL)	*t*_max_ (h)	AUC_0-last_ (ng·h/mL)	AUC_0-inf_ (ng·h/mL)	*t*_1/2_ (h)
*Group 1 (90 mg/m*^*2*^*)*					
* N*	6	6	6	6	6
Mean	9809	0.18	4707	4708	0.43
SD	3418	0.03	1732	1732	0.11
%CV	34.8	18.8	36.8	36.8	26.0
*Group 2 (120 mg/m*^*2*^*)*					
*N*	6	6	6	6	6
Mean	16256	0.18	8242	8244	0.50
SD	4434	0.06	2794	2796	0.07
%CV	27.3	34.7	33.9	33.9	14.5

*C*_*max*_ maximum concentration, *t*_*max*_ time of maximum observed concentration, *AUC*_*0-last*_ area under the concentration–time curve from the time of dosing to the time of the last measurable (positive) concentration, *AUC*_*0-inf*_ area under the concentration–time curve from the time of dosing extrapolated to infinity, *t*_*1/2*_ elimination half-life, *N* number of patients, *SD* standard deviation, *CV* coefficient of variation

## Discussion

This multicenter, open-label, phase I/II clinical study of rapid BDM afforded the following results. Regarding primary endpoints, bendamustine 90 mg/m^2^/day did not cause any new safety signals in Group 1, and bendamustine 120 mg/m^2^/day was well tolerated by Group 2 patients. Regarding secondary endpoints, plasma bendamustine concentrations peaked at the end of infusion in Groups 1 and 2, as described in the highlights of prescribing information on BENDEKA^®^, ready-to-dilute (RTD) injectable liquid formulation of bendamustine hydrochloride [13], followed by rapid elimination in a triphasic manner after the last dose, as observed in the simulation model presented by Owen et al. [11], and bendamustine 90 mg/m^2^/day was effective for Group 1 patients. The pharmacokinetic parameters (*C*_max_, *t*_max_, and AUC_0-last_) of bendamustine 90 and 120 mg/m^2^ in the present study were similar to those obtained with the same dose of original BDM in previous studies conducted by Ogura et al. [3, 22]. Furthermore, the pharmacokinetic parameters (*C*_max_, AUC_0-last_, and AUC_0-inf_) tended to be higher in Group 2 than in Group 1, and exposure increased along with an increase in bendamustine dose. No major differences in pharmacokinetic parameters were found for the 10-min dosing used in the present study or in the clinical study conducted by Cheung et al. [10]. Therefore, this clinical study in Japanese patients with iNHL, MCL, or rrDLBCL provides clinical evidence about the safety and tolerability of rapid BDM, as did the clinical study of Cheung et al. [10].

Ogura et al. conducted a phase I and pharmacokinetic study of original BDM 90 and 120 mg/m^2^ in 8 Japanese patients with relapsed or refractory iNHL and in 1 Japanese patient with relapsed or refractory MCL [3]. The AUCs for bendamustine in their study were 8.3 ± 3.6 and 10.2 ± 5.8 μg·h⁄mL in patients receiving 90 and 120 mg⁄m^2^, respectively, compared to the respective AUC_0-last_ of 4707 ± 1732 and 8242 ± 2794 ng·h/mL in the present study. Original BDM was safe for and well tolerated by the studied patients, and 120 mg/m^2^ was the recommended dose for a phase 2 clinical trial. The present study showed efficacy results similar to those (CR rates of 67.8% and 70.0% for iNHL and MCL, respectively) from phase 2 clinical study of original BDM 90 mg/m^2^/day and rituximab 375 mg/m^2^ in Japanese patients with treatment-naïve iNHL or MCL conducted by Ogura et al. [20] and those (CR rates of 40% and 30% for the bendamustine plus rituximab group and the R-CHOP group, respectively) from a phase 3 noninferiority study in patients with iNHL and mantle-cell lymphomas conducted by Rummel et al. [23].

The present clinical study of rapid BDM was not designed to examine the bioequivalence of original BDM and rapid BDM in relevant Japanese patients, because Cheung et al. conducted a phase 1, open-label, randomized, crossover study [10] to strictly evaluate the pharmacokinetics of these two formulations of bendamustine, demonstrating that they are bioequivalent and that rapid BDM is associated with a lower incidence of TEAEs than original BDM, except for abdominal pain, dehydration, pyrexia, and dyspnea. Nevertheless, the study exhibited a moderate gap from treatments in real-world clinical settings due to the small number of patients. The authors admitted the need to conduct additional studies to confirm the lower incidence of TEAEs observed with rapid BDM and recognized some limitations of the open-label study regarding the determination of bioequivalence due to a reduced population of evaluable patients in real-world oncology clinical care settings. The present study provides data on the long-term safety of rapid BDM that are supplementary to the above study and PK data in Japanese patients with iNHL, MCL, or rrDLBCL.

In consideration of the efficacy, safety, tolerability, and bioequivalence of original BDM and rapid BDM in the United States and based on the similar pharmacokinetic data for the 10- and 60-min infusion formulations obtained inside and outside Japan, given the similar safety profiles between original BDM and rapid BDM already in use in the US, we do not see any particular medical concern arising in the clinical use of rapid BDM to treat Japanese patients with hematologic malignancies, and we believe that the present study shows evidence of the benefits of rapid BDM for patients and clinicians.

In Japan, the original BDM is reconstituted with saline to prepare a 250-mL admixture for 60-min dosing. Rapid BDM, which allows for 10-min dosing of an admixture, shortens infusion time and causes an approximately 80% reduction in the volume of normal saline required to prepare the admixture. Thus, rapid BDM is expected to be beneficial for both patients and medical professionals, because the risk of edema or extravasation in patients is lessened and the electrolyte load of patients with impaired renal function is attenuated by a reduced volume of fluid. Furthermore, the patient’s stress is reduced, and the work of medical professionals who prepare and infuse the admixture is ameliorated by the shortened infusion time, leading to operational streamlining and cost reductions in the hospital or clinic.

In conclusion, rapid BDM shows good safety, tolerability, and efficacy in Japanese patients with previously untreated iNHL or MCL and in those with rrDLBCL. Rapid BDM has the potential to shorten the setup time for bendamustine infusion and to significantly reduce the treatment burden of patients and health care providers. Hence, rapid BDM may be a useful therapeutic alternative to the currently available 60-min dosing of bendamustine with benefits by simplifying outpatient treatment.

## Supplementary Information

Below is the link to the electronic supplementary material.

Supplementary file1 (DOCX 17 KB)
