# Supplementary material for: A phase I/II study of 10-min dosing of bendamustine hydrochloride (rapid infusion formulation) in patients with previously untreated indolent B-cell non-Hodgkin lymphoma, mantle cell lymphoma, or relapsed/refractory diffuse large B-cell lymphoma in Japan
Source: Cancer Chemother Pharmacol. 2022 Jul 7;90(1):83–95. doi: 10.1007/s00280-022-04442-2 (PMC9300521; doi:10.1007/s00280-022-04442-2)
Supplement: Supplementary file 1 — Supplementary file1 (DOCX 17 KB) [file 280_2022_4442_MOESM1_ESM.docx]

Supplementary Table 1 Pharmacokinetic parameters obtained in the present study and

the US study

| Study | Subject  (dose) |  | C_max_  (ng/mL) | t_max_  (h) | AUC_0-last_  (ng・h/mL) | AUC_0-inf_ (ng・h/mL) | t_1/2_ (h) |
| --- | --- | --- | --- | --- | --- | --- | --- |
| Present study | Group 2 patients (120 mg/m^2^) | N | 6 | 6 | 6 | 6 | 6 |
|  |  | Mean | 16256 | 0.18 | 8242 | 8244 | 0.50 |
|  |  | %CV | 27.3 | 0.1-0.3* | 33.9 | 33.9 | 14.5 |
| US study^[10]^ | Patients evaluable for PK (120 mg/m^2^) | N | 38 | 38 | 38 | 38 | 38 |
|  |  | Mean | 19158 | 0.18 | 10339 | 10370 | 0.65 |
|  |  | %CV | 34 | 0.1-0.4* | 49 | 49 | 37.3 |

*: Range

PK, pharmacokinetics; N, number of patients; CV, coefficient of variation
